# The Common Accidents of Every Day Life

**Published:** 1887-11-12

**Authors:** Robert A. P. Forrester


### THE COMMON ACCIDENTS OF EVERY-DAY LIFE.

By Robert A. P. Forrester, B.A.Dub., M.B. and C.M.Edin.

### INFANCY.

" First the infant, mewling and puking in his nurse's arms."

(*Continued from p.* 76.)

### Concussion of Spine.

Some few years ago an extraordinary accident happened to a young relative of the writer's, fortunately attended with no serious result. The child, a little girl about eighteen months old, was out in a perambulator in charge of its nurse. During the course of the walk they came to a prettily-wooded road, which at one part was only about twenty yards from the edge of a quarry, where there was no railing or protection of any kind. The nurse having met an acquaintance, and being more interested in the conversation that ensued than attentive to her charge, did not perceive that the perambulator was moving down the grassy slope towards the edge of the cliff, and gaining in speed at every foot. A cry from the child caused her to turn round, and to her horror she perceived the carriage and child disappear over the edge. She ran down, and without considering where she was going jumped after it. The child being strapped in the perambulator did not fall out, although the carriage and its contents turned a somersault, and after falling a distance of nearly thirty feet, alighted on a soft mound of loose earth and sand at the bottom of the quarry. The nurse landed on a ledge further up, none the worse for her jump. On examination of the child no injury of any kind was made out, nor was the perambulator broken in any way.

Almost any injury might have occurred, *e.g.*, broken neck, broken limbs, concussion of spine, brain, etc., etc.

In the event of a nurse letting a young child fall on its back, what would lead one to suspect such an injury as concussion? A few words regarding the spinal column and cord will help to make this point clear. The vertebral or spinal column or backbone consists of a number of superimposed bones or vertebræ, which move and turn somewhat on each other, and extends from the head to the haunch bones. The vertebræ in young children are thirty-three in number, but in the adult, on account of the fusion of the two lowest groups, they are reduced to twenty-six. Each vertebra may be said to consist of two parts, a centre or body, and an arch. The body is a short cylinder, from the back of which the arch springs. This consists of two plates of bone united behind, and enclosing the spinal cord, or nervous axis. Between the vertebræ are pads of gristle, which act as buffers, preventing the bones jarring when they move, and giving elasticity to the whole column. It is this gristle or cartilage which to a certain extent becomes absorbed in old age, accounting for the decrease in stature at that period of life. The bones are kept in position by strong bands of fibrous tissue, which pass round them in every direction. It will be seen, therefore, that the rings or arches of bone which spring from each vertebral body form collectively a very complete protecting canal for the spinal cord. The bones in the column are arranged in groups, and named from their position; *e.g.*, those in the neck are called cervical, and are seven in number; lower down we have the twelve dorsal vertebræ, to which the ribs are attached; then the four lumbar or loin vertebræ. These with the cervical have the freest range of movement, as anyone may prove for himself by bending the neck or the body. Next come the sacral, which form one bone in the adult, although in the child there were five; and lastly, the coccygeal, or tail vertebræ, also forming one bone in the adult, but originally four. The two last bones are called *false* vertebræ, in contradistinction to the others, or *true* vertebræ. The spinal cord occupies the spinal canal, as described, and extends from the large opening in the base of the skull, called *foramen magnum*, to the body of the first lumbar vertebra. Its length varies from fifteen to eighteen inches. This nervous cord we may call the central telegraphic wire of the body, which sends off numerous branches through the intervertebral openings all over the body, and along it messages to and from the brain are transmitted. It is evident, therefore, that any injury to this important structure will affect parts which receive their nervous supply from *below* the seat of the injury, simply because the communication between the brain, or telegraphic office, and those parts has been interrupted. In some cases messages can no longer be sent either way, or, if sent, are imperfectly understood.

We mentioned *Concussion*, which simply means a *shaking together*, and may be produced by such an accident as the above, or by a person being forcibly projected forwards and then suddenly forced backwards, as in a railway accident. Such a shake or jar is apt to derange the organic structure of the cord, and interfere with its functions.

The symptoms of Concussion vary somewhat according to the situation of the injury, the violence of the blow, etc. A blow in the cervical region may cause instant death, a less severe one paralysis of one or more limbs. In the dorsal or lumbar regions paraplegia, or paralysis of both legs may be induced.

It will generally be found, however, that the subject of such an injury suffers from shock, nausea, and perhaps vomiting, feeble and intermittent pulse, and with occasional twitching of the limbs. *Shock* is defined by Sir William MacCormac as "a condition of sudden depression of the whole of the functions of the body," manifested by sickness, fainting, coldness of the surface, pallor, sweatings, weakness, and irregularity of the pulse and breathing, shakings, and sometimes unconsciousness.

With the history of a fall and the presence of the above symptoms, or only some of them, the presumption will be that some injury has occurred to the spinal cord, probably Concussion. On account of the obscurity and slightness at first of some of the symptoms this may not be diagnosed at the time. What are we to do?

Remember that the *immediate* treatment of a badly-injured person has great influence, not only in the early stages of the progress of the case, but also on the whole result.

Judicious and intelligent management *at first* may ensure a safe recovery, whilst the contrary may have an opposite effect.

Get an exact history of the accident, if possible. Examine the patient carefully and gently, but *do not raise him* at first, as he may be suffering from shock. If no limbs are broken, no injury to the head or trunk, it will be advisable to combat the shock in the first instance. This is best done by the application of warmth, and possibly stimulants. Get the little patient as soon as possible between warm blankets, and apply hot bottles wrapped in flannel to the trunk and limbs, warm linseed-meal poultices or turpentine stupes to the chest; and, if he can swallow, a teaspoonful of weak, warm brandy and water may be given. Ammonia to the nostrils is often useful.

After the symptoms of shock have subsided the treatment consists in obtaining absolute rest of both mind and body, and, unless the shock has been very severe, improvement may be looked for in a very few days. It sometimes happens that nurses, in fear of the consequences, have concealed the occurrence of such an accident from the parents, and the child has probably been moved about as usual immediately after the first effects have passed away. Unless the patient obtains rest and quiet for some time there is danger of disease of the spinal cord being subsequently developed.

